# The Biomedical Publications Industry Must Change to Better Serve the Needs of Science and Scientists

**DOI:** 10.20411/pai.v10i2.819

**Published:** 2025-03-31

**Authors:** Michael M. Lederman, Neil S. Greenspan

**Affiliations:** 1 Case Western Reserve University School of Medicine, Cleveland, Ohio

**Keywords:** Biomedical Publications, Publication Fees, Scientific Publications, Journal Formatting, Open Access, Peer Review

## Abstract

The biomedical publications industry is vital to progress in science and health care. We observe that this industry has become unnecessarily complex and expensive for researchers and readers, impeding the sharing of research findings. In this perspective, we offer selected critiques of this industry and suggest how it might be improved.

## INTRODUCTION

The biomedical research publications industry plays a crucial role in the documentation and dissemination of research progress that has profound impact on scientific knowledge, understanding, and health care. Thus, it is important that this industry assures accuracy, maintains high standards of integrity, is accessible to researchers and the public, and that its operations are efficient, thereby supporting timely review and publication of important findings. Here, we offer selected critiques of this industry and suggest how it might be improved.

The relationship between researchers and biomedical publications is imbalanced. While the industry depends entirely on researchers to generate their product, researchers willingly compete to have their studies appear in the most prestigious journals to enhance their reputations, promote their advancement, and attract competitive research support. Despite this mutual dependence, most journals sustain submission, review, and publication policies that may serve the interests of the journals but are wasteful of researchers' time, energy, and resources and offer less benefit to the disciplines they purport to serve than might be desired.

Below, we delineate examples of journal policies, procedures, and practices that are sub-optimal. We also provide, where possible, alternative policies, procedures, or practices that reduce the time, energy, and effort that must be invested by researchers to achieve their aims in disseminating their findings and insights.

## DISTRIBUTION OF FINANCIAL GAIN

The biomedical research industry can be very profitable. Last year, Elsevier made a profit of 1.79 billion pounds on revenue of 9.16 billion pounds [1].

Many corporate publishers of biomedical research are similarly well-resourced. Nevertheless, some journals run by these enterprises charge thousands of dollars for the publication of accepted manuscripts. Yet these same organizations depend upon researchers to serve as reviewers without direct compensation.

## SUBMISSION PROCESSES

Authors target journals that publish works in their field or more general journals with high visibility that are broadly read and respected. Most journals ask that works submitted for consideration follow a particular format that may be unique for that journal or may be shared by a handful of others. If the work is rejected by the authors' first-choice journal, submission to another generally follows. Submission is often delayed because the second journal format usually differs from the first. This is time-consuming and rarely necessary to determine if the work has merit.

A recent analysis of submissions policies of more than 300 leading biomedical publications estimated that approximately 230 million US dollars were lost in 2021 due to costs of researchers reformatting articles after a first rejection [2]. One solution is to have an Open Format policy for submissions, eliminating the time waste of reformatting for each submission. Since its launch in 2016, *Pathogens and Immunity* reviews submissions in any legitimate format and requires revision to its format only if the piece is accepted. A number of other journals have also adopted this policy, and the number appears to be growing.

Most scientific journals support online submissions of manuscripts. This process is typically time-consuming and complicated, often requiring that all authors sign statements defining their role in the research and manuscript preparation and reporting all conflicts of interest. We assume that journal publishers fear mis-statements regarding these important issues and have decided that these requirements somehow protect the publisher from an ill-defined liability. The editors of *Pathogens and Immunity* believe that the benefits of reducing such requirements will on balance benefit journals and authors, which is why we require only that the submitting author state that all authors have contributed to the work, that their contributions are defined, that all relevant conflicts of interest are stated, that all masthead authors have approved the manuscript, and that the work is not under consideration for publication elsewhere.

We recognize that this looser policy could allow errors of authorship to appear, but in the past 8 years, only one has been identified and was rapidly corrected. This was a failure by the submitting author to list a contributing author that would not have been identified by any journal authorship policies of which we are aware.

We support making manuscript submission as painless as possible such that it could be completed in less than 5 minutes. We don't know what is done with the detailed information requested by many journals but suspect it is used by some to generate lists of potential reviewers, subscribers, recipients of advertisements and other solicitations. We don't know how useful this information is to more selective journals and suggest that if it is wanted, a more efficient approach would be to request it only after acceptance.

## REVIEW OF SUBMISSIONS

Typically, an editor will determine, either alone or after consultation with other editors, whether the work should be sent out for formal review. High-quality reviews are necessary to assure integrity of the publication process, and editors often struggle to find experts willing to evaluate a study. Academic tradition values peer review in one's field that may offer the potential benefit of early exposure to valuable insights, but careful reviews take time that is often in short supply. While some publishers offer incentives such as discounts on submission or publication fees that are somewhat self serving, these discounts are a fraction of the typical charges to authors for publication. *Pathogens and Immunity* provides a modest ($50) honorarium to all reviewers who provide a timely review. The cost is worth it and may serve as a respectful incentive for reviewers such that *Pathogens and Immunity* provides rapid first decisions (median 14 days from submission to the first editor's decision) to authors.

## REVIEW QUALITY

One approach to encourage the quality and fairness of reviews is to evaluate them. At *Pathogens and Immunity*, senior editors are expected to rate the quality of each review. This helps guide selection of reviewers for future submissions. Top scored reviewers receive written notice of their evaluation (and a token gift) at year's end. In the next calendar year, we are considering including the senior editors' scores of the review to each reviewer. We are open to additional suggestions from other editors and scientists.

## COST

Costs of publication are typically recovered by institutional and personal subscription fees to readers, submission and publication fees to authors, fees for access to single archived publications, and advertising charges. Open Access resolves costs to readers but often comes with publication charges of thousands of dollars to researchers that can be daunting particularly to young authors and those in less wealthy institutions. *Pathogens and Immunity* is Free Open Access to all readers, charges no fees to authors for submission or publication, and assigns copyright to authors but recognizes that enforcement of copyright is not practicable. The journal contains no paid advertisements. *Pathogens and Immunity* can maintain these policies as it is fully supported by a generous endowment from a charitable foundation (The Fasenmyer Fund) that is managed by our university.

While this may not be an option for most existing journals, universities, their donors and charitable agencies are positioned to develop high-quality author- and reader-friendly research journals at not much more than the cost of endowing a senior faculty chair (as Fasenmyer and Case Western Reserve University did). Other academic faculty are already moving in a similar direction aiming to improve the environment of academic research, with some contending that the for-profit publications industry (whether open access or not) has had a deleterious impact on their fields of inquiry [3].

## WHAT CAN EXISTING JOURNALS DO?

Although we encourage universities and charitable agencies to fund and support a new model of scientific publication, we are not condemning for-profit publications, but rather, we suggest that scientific publication can be sculpted to better serve the interests of science, authors, and readers. Our experience offers a menu of policies and strategies, many of which can be implemented at minimal or no cost even by for-profit journals.
